# Complementary and alternative medicine use among persons with multiple chronic conditions: results from the 2012 National Health Interview Survey

**DOI:** 10.1186/s12906-018-2342-2

**Published:** 2018-10-19

**Authors:** Justice Mbizo, Anthony Okafor, Melanie A. Sutton, Bryan Leyva, Leauna M. Stone, Oluwadamilola Olaku

**Affiliations:** 1 Department of Public Health, University of West Florida, 11000 University Parkway, Bldg. 38/Room 127, Pensacola, FL 32514 USA; 2 Department of Mathematics and Statistics, University of West Florida, Pensacola, FL USA; 3 Warren Alpert Medical School, Brown University, Providence, RI USA; 4 Office of Cancer Complementary and Alternative Medicine, National Cancer Institute, Bethesda, MD USA; 5 Kelly Government Solutions, Bethesda, MD USA

**Keywords:** Complementary and alternative medicine, Chronic disease, Comorbidity, Diabetes, Hypercholesterolemia, Hypertension, Integrative medicine, Obesity

## Abstract

**Background:**

Although a quarter of Americans are estimated to have multiple chronic conditions, information on the impact of chronic disease dyads and triads on use of complementary and alternative medicine (CAM) is scarce. The purpose of this study is to: 1) estimate the prevalence and odds of CAM use among participants with hypercholesterolemia, hypertension, diabetes, and obesity; and 2) examine the effects of chronic condition dyads and triads on the use of CAM modalities, specifically manipulative and body-based methods, biological treatments, mind-body interventions, energy therapies, and alternative medical systems.

**Methods:**

Data were obtained from the 2012 National Health Interview Survey and the Adult Alternative Medicine supplement. Statistical analyses were restricted to persons with self-reported hypercholesterolemia, hypertension, diabetes, or obesity (*n* = 15,463).

**Results:**

Approximately 37.2% of the participants had just one of the four chronic conditions, while 62.4% self-reported multiple comorbidities. CAM use among participants was as follows (*p < 0.001*): hypercholesterolemia (31.5%), hypertension (28.3%), diabetes (25.0%), and obesity (10.8%). All combinations of disease dyads and triads were consistently and significantly associated with the use of mind-body interventions (2–4%, *p < 0.001*). Two sets of three dyads were associated with use of manipulative methods (23–27%, *p < 0.05*) and energy therapies (0.2–0.3%, *p < 0.05*). Use of biological treatments (0.04%, *p < 0.05*) and alternative systems (3%, *p < 0.05*) were each significant for one dyad. One triad was significant for use of manipulative methods (27%, *p < 0.001*).

**Conclusions:**

These findings point to future directions for research and have practical implications for family practitioners treating multimorbid patients.

## Background

The National Center for Complementary and Integrative Health, the federal government’s lead agency for scientific research on complementary and alternative medicine (CAM), defines CAM as a category of medicine that includes a variety of treatment approaches that fall outside the realm of conventional medicine. CAM therapies have been grouped into five distinct domains: manipulative and body-based methods, biological treatments, mind-body interventions, energy therapies, and alternative medical systems [1]. Evidence from national studies suggest that approximately one-third of Americans used some form of CAM in the past 12 months [2]. Use of non-conventional medicine in the general population has been increasing in the past decade and contributes substantially to health care spending. In 2007, Americans spent nearly $34 billion in out-of-pocket expenses on CAM, which represented a 25% increase in spending compared earlier estimates from 1997 trends [3].

A substantial body of research has focused on understanding trends and patterns of CAM use in the general U.S. population [2, 4], as well as among specific patient subgroups. Several studies have examined correlates and predictors of CAM use among patients with specific chronic diseases, including diabetes [5], cancer [6], and cardiovascular disease [7], with evidence suggesting high CAM use among patients with chronic illness. Despite advances in the collective understanding of CAM use among patients with chronic illness, little is known about the utilization of CAM among patients with multiple chronic conditions. Although more than a quarter of Americans are estimated to have multiple comorbidities, we identified few studies [8] assessing the effect of specific combinations of chronic diseases (dyads and triads) on use of CAM. Notably, none of these studies focused specifically on patients with cardiovascular risk factor combinations such as hypertension, hypercholesterolemia, diabetes, and obesity, all of which are conditions of increasing prevalence and public health significance. Whereas the prevalence of hypertension among adults in the U.S. has remained relatively constant at 30% since 1999 [9–11], the prevalence of diabetes (9–12%), hypercholesterolemia (25–27%), and obesity (31–36%) has been steadily increasing in the past decade [9].

Given the increasing prevalence of these risk factors and their significant contributions to cardiovascular morbidity and mortality in the U.S. [12] and around the world, this is an important gap in the scientific literature. To address this gap, this study sought to: 1) estimate the prevalence and odds of CAM use among participants with major chronic diseases such as hypercholesterolemia, hypertension, diabetes, and obesity; and 2) examine the effects of chronic condition dyads and triads on the use of specific CAM modalities and treatments. We anticipate findings from this study may be useful in generating hypotheses for future research and have practical implications for family physicians and chronic disease self-management specialists.

## Methods

### Study design and participants

This cross-sectional study consists of men and women ages 18 years and older who responded to the 2012 National Health Interview Survey (NHIS), a nationally-representative surveillance system administered by the Centers for Disease Control and Prevention’s National Center for Health Statistics (NCHS). The NHIS serves as “the principal source of information on the health of the civilian non-institutionalized population in the United States.” [13] Beginning in 2002, the NCHS added a supplementary module on CAM utilization that is administered every five years. This study specifically focused on persons with self-reported hypercholesterolemia, hypertension, diabetes, or obesity (*n* = 15,463). The NHIS survey is a population-based surveillance system administered by the U.S. Centers for Disease Control and Prevention. The survey consists of face-to-face household interviews to obtain data for each respondent selected to complete the NHIS instrument and is administered by NCHS trained interviewers. To ensure a nationally representative data set for the civilian noninstitutionalized population of the United States, a multistage stratified sampling design is used, with interviews conducted in all 50 states and the District of Columbia. Computer-assisted data collection is utilized during each interview to perform data quality checks as responses are made and to ensure the consistency of the questionnaire flow.

The NHIS survey has several components broken down into modules. The Household module provides information on household composition and survey response characteristics. The Family module provides information on participants’ relationships and family structure within households. Individual information is contained in the Person and Sample Adult modules. The Person module contains information on individual health status, health care access and utilization, health insurance, basic socio-demographics, income/assets, and family food security. The Sample Adult module contains questions on socio-demographics, health conditions/status, health behaviors, and health care access and utilization administered to randomly selected adults within the family. Finally, the Adult Alternative Medicine module provides information on the adult sample use of non-conventional health care practices. A detailed discussion of the NHIS instrument is described elsewhere [14, 15].

### Measures

Overall CAM use was measured by collapsing all reported CAM products into the five domains noted previously: [1] (1) manipulative and body-based methods, including chiropractic/osteopathic approaches and massage therapy; (2) biological treatments, including herbal remedies and special diets; (3) mind-body interventions, including meditation, hypnosis, prayer, and art/music therapy; (4) energy therapies, including biofield and bioelectromagnetic-based therapies, and (5} alternative medical systems, including acupuncture, Ayurvedic medicine, homeopathy, and naturopathy. A composite variable for CAM use was created by combining the domains in which CAM use was present if any one of the five domains was coded as *“1”,* indicating reported CAM use within that domain. The final CAM variable was dichotomized into “0” and *“1”,* where *“0”* represented absence of CAM use. The independent variables included socio-demographic and socio-economic characteristics such as age, sex, race/ethnicity, education, marital status, family income, insurance coverage, having a regular source of care, and region of residence.

### Statistical analysis

The analysis consisted of descriptive statistics, bivariate analysis, and multivariate logistic regression. The descriptive analysis included counts, means, standard deviations, and proportions of CAM use. For the bivariate analysis, we performed a Chi-square test of independence to assess the association and significance of each covariate and CAM use. The multivariate logistic regression method, a critical component of the analysis, was used to assess the association between the dichotomous response variable describing CAM use and the predictor variables or covariates [16]. To account for the confounding effect of the covariates in the multivariate analysis, the adjusted odds ratios (aOR) were calculated. The 95% confidence intervals for the odds ratio were determined and used not only to assess the significance of the covariates but also to determine the magnitude of the effect based on the range of the interval.

All analyses were performed using Stata 15 for Windows (STATA Corp., College Station, Texas). Data from the NHIS Family, Household, Person, Sample Adult, and Adult Alternative Medicine files were merged, and sampling weights were applied to account for the complex probability survey design [13]. Using the multivariate and bivariate analysis previously described, we further stratified the analysis by disease dyads and triads across the five domains of CAM and examined the associations between the different chronic disease dyads and triads with respect to specific CAM modality use. Statistical significance was set at a *p*-value of less than 0.05.

## Results

Table 1 presents descriptive statistics and the frequency of the four chronic conditions by covariates. Overall CAM use proportions in general, CAM use in participants with two or more chronic conditions, and a comparison of the adjusted odds of CAM use for those participants with one chronic condition versus two or more conditions are presented in Table 2 Table 3 provides the bivariate proportions for CAM use among disease dyads and triads for the five CAM domains. Finally, Table 5 summarizes statistically significant CAM domain use by disease status, including individual chronic diseases, as well as dyads and triads.

**Table 1:** Characteristics of the study sample and frequency of chronic conditions, national health interview survey, 2012

Characteristics		Chronic Condition			
	Overall Sample	Hypercholesterolemia	Hypertension	Diabetes	Obesity
	n(%)	n(%)	n(%)	n(%)	n(%)
Age Group					
< 35	1241(8.0)	502(39.9)	823(66.6)	137(11.1)	490(39.8)
35–49	2946(18.8)	1658(56.4)	1860(63.1)	516(17.5)	1327(44.9)
50–64	5479(35.5)	3496(63.2)	3966(72.5)	1338(24.0)	2287(41.7)
> 64	5797(37.7)	3833(66.0)	4649(80.2)	1537(26.3)	1523(26.4)
Sex					
Male	6964(44.9)	4322(61.8)	5045(72.5)	1616(22.9)	2503(35.9)
Female	8499(55.1)	5167(60.5)	6253(73.7)	1912(22.3)	3124(36.8)
Race and Ethnicity					
Non-Hispanic White	11,663(75.6)	7423(63.4)	8212(70.5)	2500(21.3)	4186(35.8)
Non-Hispanic Black/African American	2746(17.6)	1377(49.7)	2341(85.6)	762(27.5)	1199(44.2)
Alaska Natives/American Indians	158(1.0)	86(54.1)	122(78.9)	53(33.7)	76(50.8)
Non-Hispanic Other	896(5.8)	603(67.4)	623(69.5)	213(23.3)	166(18.3)
Education Level					
Less than High School	2890(18.5)	1718(59.1)	2291(79.6)	858(29.5)	1062(36.8)
High School Graduate/Some College	8877(57.5)	5358(60.1)	6589(74.3)	2060(22.9)	3445(38.7)
College/Professional Graduate	3696(24.0)	2413(65.2)	2418(65.4)	610(16.7)	1120(30.5)
Marital Status					
Married	8131(52.7)	5219(64.0)	5606(68.9)	1778(21.8)	2996(37.0)
Widowed/Divorced	5114(33.1)	3150(61.2)	4052(79.4)	1293(24.7)	1682(32.7)
Single/Never Married	2218(14.2)	1120(49.9)	1640(74.3)	457(20.5)	949(42.8)
Family Income					
< $20,000	6696(43.2)	3904(58.2)	5322(79.6)	1850(27.2)	2566(38.2)
$20,000–$34,999	2107(13.8)	1312(62.0)	1546(73.4)	460(21.9)	802(38.0)
$35,000–$49,999	2306(14.9)	1448(62.2)	1597(69.4)	473(21.0)	839(36.6)
$50,000–$74,999	3435(22.3)	2256(65.3)	2145(62.5)	544(15.6)	1173(34.2)
$75,000+	919(5.8)	569(61.7)	688(75.4)	201(21.3)	247(26.9)
Health Insurance Coverage					
Yes	13,742(88.8)	8655(62.6)	10,009(73.0)	3198(23.0)	4894(35.7)
No	1721(11.2)	834(48.9)	1289(74.7)	330(19.3)	733(42.2)
Regular Source of Care					
Yes	14,174(91.6)	8836(62.1)	10,389(73.4)	3353(23.4)	5240(37.0)
No	1289(8.4)	653(50.7)	909(70.3)	175(13.5)	387(30.1)
Geographic Region					
Northeast	2554(16.6)	1610(62.4)	1803(70.8)	545(20.9)	878(34.2)
Midwest	3206(20.8)	1981(61.4)	2320(72.2)	694(21.3)	1244(39.0)
South	5962(38.6)	3581(59.9)	4536(76.3	1478(24.8)	2276(38.4)
West	3741(24.0)	2317(61.9)	2639(70.6)	811(21.4)	1229(32.5)
Hypercholesterolemia					
Yes	9489(61.1)	–	5853(61.8)	2219(23.1)	3390(35.8)
No	5974(38.9)	–	5445(91.1)	1309(21.8)	2237(37.3)
Hypertension					
Yes	11,298(73.2)	5853(51.6)	–	2591(22.7)	4422(39.1)
No	4165(26.8)	3636(87.1)	–	937(22.4)	1205(28.9)
Diabetes					
Yes	3528(22.6)	2219(62.4)	2591(73.4)	–	1722(48.9)
No	11,935(77.4)	7270(60.7)	8707(73.1)	–	3905(32.8)
Body Mass Index					
< 18.5 (Underweight)	782(5.1)	451(57.8)	579(73.5)	178(23.0)	–
18.5–24.9 (Normal)	3618(23.6)	2199(60.2)	2461(68.0)	502(13.9)	–
25.0–29.9 (Overweight)	5436(34.9)	3449(63.2)	3836(70.8)	1126(20.4)	–
30.0+ (Obese)	5627(36.4)	3390(60.1)	4422(78.7)	1722(30.4)	–
Manipulative Methods					
Yes	3778(24.3)	2492(65.7)	2678(71.1)	762(20.2)	1425(38.0)
No	11,685(75.7)	6997(59.6)	8620(73.8)	2766(23.4)	4202(35.9)
Mind-Body Interventions					
Yes	801(5.1)	497(62.0)	518(64.9)	88(11.4)	220(27.7)
No	14,662(94.9)	8992(61.1)	10,780(73.6)	3440(23.2)	5407(36.9)
Biological Treatments					
Yes	42(0.3)	24(58.7)	32(76.2)	7(15.9)	11(27.0)
No	15,421(99.7)	9465(61.1)	11,266(73.2)	3521(22.6)	5616(36.4)
Energy Therapies					
Yes	72(0.5)	49(67.3)	38(51.8)	11(14.5)	29(39.1)
No	15,391(99.5)	9440(61.1)	11,260(73.3)	3517(22.6)	5598(36.4)
Alternative Systems					
Yes	592(3.8)	395(66.9)	394(66.6)	97(16.5)	204(34.8)
No	14,871(96.2)	9094(60.9)	10,904(73.4)	3431(22.8)	5423(36.5)

Note: All frequencies are unweighted; all percentages are weighted

**Table 2:** Proportions and odds of CAM use among adults with and without comorbidities

Independent Variables	Overall CAM Use		CAM Use in Patients with 2 or More Chronic Conditions		Adjusted Odds of CAM Use			
					Patients with 1 Chronic Condition		Patients with 2 or More Chronic Conditions	
	n(%)	*χ*^*2*^, *p*-value	n(%)	*χ*^*2*^, *p*-value	aOR [95% CI]	*p*-value	aOR [95% CI]	*p*-value
Age Group		6.69, *p* < 0.001		14.04, *p* = 0.003				
< 35 (ref)	349(28.3)		156(26.9)		1.00	–	1.00	–
35–49	887(29.9)		473(27.7)		1.12[0.89,1.39]	0.331	1.04[0.83,1.30]	0.729
50–64	1740(31.6)		1108(31.0)		1.07[0.86,1.32]	0.548	1.21[0.98,1.49]	0.083
> 64	1607(27.6)		1042(27.4)		0.94[0.75,1.18]	0.529	1.10[0.88,1.37]	0.417
Sex		3.28, *p* = 0.071		2.29, *p* = 0.130				
Male (ref)	1999(28.7)		1219(28.0)		1.00	–	1.00	–
Female	2584(30.2)		1560(29.4)		1.25[1.11,1.42]	< 0.001	1.29[1.17,1.42]	< 0.001
Race and Ethnicity		79.11, *p* < 0.001		161.97, *p* < 0.001				
Non-Hispanic White (ref)	3857(32.9)		2307(32.0)		1.00	–	1.00	–
Non-Hispanic Black/African American	460(16.4)		320(17.2)		0.46[0.37,0.56]	< 0.001	0.52[0.45,0.60]	< 0.001
Alaska Natives/American Indians	39(25.2)		24(22.4)		0.98[0.52,1.85]	0.949	0.64[0.40,1.02]	0.058
Non-Hispanic Other	227(25.6)		128(25.8)		0.61[0.47,0.78]	< 0.001	0.75[0.60,0.94]	0.012
Education Level		111.49, *p* < 0.001		132.62, *p* < 0.001				
Less than High School (ref)	533(18.8)		369(19.1)		1.00	–	1.00	–
High School Graduate/Some College	2696(30.0)		1694(29.6)		1.80[1.48,2.19]	< 0.001	1.53[1.34,1.74]	< 0.001
College/Professional Graduate	1354(36.5)		716(35.3)		2.12[1.70,2.65]	< 0.001	1.78[1.51,2.09]	< 0.001
Marital Status		12.49, *p* < 0.001		16.36, *p* < 0.001				
Married (ref)	2550(31.2)		1533(30.2)		1.00	–	1.00	–
Widowed/Divorced	1465(28.3)		919(28.0)		1.04[0.90,1.21]	0.589	1.02[0.92,1.15]	0.666
Single/Never Married	568(25.9)		327(24.8)		0.96[0.79,1.16]	0.681	0.92[0.79,1.07]	0.291
Family Income		38.48, *p* < 0.001		97.68, *p* < 0.001				
< $20,000 (ref)	1652(24.7)		1097(24.6)		1.00	–	1.00	–
$20,000–$34,999	613(29.0)		367(27.3)		1.12[0.93,1.36]	0.245	0.98[0.85,1.14]	0.838
$35,000–$49,999	801(34.3)		483(34.3)		1.21[1.00,1.46]	0.044	1.30[1.12,1.50]	< 0.001
$50,000–$74,999	1255(36.2)		675(35.1)		1.21[1.01,1.46]	0.040	1.25[1.08,1.44]	0.003
$75,000+	262(28.6)		157(29.1)		1.01[0.78,1.32]	0.931	1.14[0.93,1.41]	0.208
Health Insurance Coverage		4.31, *p* = 0.039		0.05, *p* = 0.816				
Yes	4107(29.8)		2489(28.8)		1.05[0.85,1.29]	0.674	0.84[0.71,0.99]	0.041
No (ref)	476(27.2)		290(28.4)		1.00	–	1.00	–
Regular Source of Care		9.36, *p* = 0.002		7.02, *p* = 0.008				
Yes	4256(29.9)		2624(29.0)		1.06[0.86,1.31]	0.596	1.09[0.88,1.35]	0.424
No (ref)	327(25.3)		155(24.1)		1.00	–	1.00	–
Geographic Region		33.08, *p* < 0.001		157.90, *p* < 0.001				
Northeast (ref)	702(27.3)		389(25.0)		1.00	–	1.00	–
Midwest	1140(35.6)		723(35.8)		1.21[1.00,1.46]	0.046	1.64[1.41,1.90]	< 0.001
South	1441(24.1)		895(23.1)		0.91[0.76,1.08]	0.268	1.03[0.89,1.19]	0.681
West	1300(34.4)		772(34.6)		1.21[1.01,1.45]	0.037	1.61[1.38,1.87]	< 0.001
Hypercholesterolemia		45.22, *p* < 0.001		13.64, *p* < 0.001				
Yes	2995(31.5)		2105(29.7)					
No	1588(26.4)		674(25.9)					
Hypertension		27.19, *p* < 0.001		0.33, *p* = 0.566				
Yes	3210(28.3)		2357(28.6)					
No	1373(32.7)		422(29.4)					
Diabetes		41.12, *p* < 0.001		25.62, *p* < 0.001				
Yes	877(25.0)		814(25.4)					
No	3706(30.8)		1965(30.4)					
Body Mass Index		12.01, *p* < 0.001		22.60, *p* < 0.001				
< 18.5 (Underweight)	158(1.0)		67(19.8)					
18.5–24.9 (Normal)	1092(7.0)		338(25.6)					
25.0–29.9 (Overweight)	1669(10.7)		712(29.6)					
30.0+ (Obese)	1664(10.8)		1662(29.6)					

Note: All frequencies are unweighted; all percentages are weighted

**Table 3:** Utilization of CAM modalities by chronic disease dyads and triads

Disease Combinations	Overall CAM Use (n = 15,463)		CAM Modalities									
			Manipulative Methods (*n* = 3778)		Biological Treatments (*n* = 42)		Mind-Body Interventions (*n* = 801)		Energy Therapies (*n* = 72)		Alternative Systems (*n* = 592)	
	(%)	*χ*^*2*^ value, *p*-value	*(%)*	*χ*^*2*^ value, *p*-value	(%)	*χ*^*2*^ value, *p*-value	*(%)*	*χ*^*2*^ value, *p*-value	*(%)*	*χ*^*2*^ value, *p*-value	*(%)*	*χ*^*2*^ value, *p*-value
Disease Dyads												
ChoBp		0.08, *p* = 0.773		5.12, *p* = 0.024		0.03, *p* = 0.872		30.13, *p* < 0.001		5.32, *p* = 0.022		1.02, *p* = 0.314
Yes	11.2		25.4		0.3		3.9		0.3		3.6	
No	18.3		23.7		0.3		5.9		0.6		4.0	
ChoDiab		6.15, *p* = 0.014		0.58, *p* = 0.447		0.25, *p* = 0.614		29.25, *p* < 0.001		4.16, *p* = 0.042		2.83, *p* = 0.094
Yes	3.8		23.7		0.2		2.6		0.2		3.1	
No	25.7		24.4		0.3		5.6		0.5		3.9	
ChoOb		10.53, *p* = 0.001		22.79, *p* < 0.001		1.27, *p* = 0.261		18.55, *p* < 0.001		2.64, *p* = 0.106		1.40, *p* = 0.238
Yes	6.9		27.4		0.2		3.7		0.7		4.1	
No	22.6		23.5		0.3		5.6		0.4		3.7	
BpDiab		17.18, *p* < 0.001		4.51, *p* = 0.035		0.15, *p* = 0.702		37.98, *p* < 0.001		4.78, *p* = 0.030		4.88, *p* = 0.028
Yes	4.3		22.7		0.2		2.6		0.2		3.0	
No	25.2		24.6		0.3		5.7		0.5		4.0	
BpOb		0.05, *p* = 0.827		2.61, *p* = 0.107		1.67, *p* = 0.197		14.34, *p* < 0.001		2.68, *p* = 0.103		3.03, *p* = 0.083
Yes	8.4		25.1		0.2		4.0		0.3		3.4	
No	21.1		24.0		0.3		5.6		0.5		4.0	
DiabOb		8.55, *p* = 0.004		2.01, *p* = 0.157		5.69, *p* = 0.018		27.74, *p* < 0.001		1.56, *p* = 0.212		2.43, *p* = 0.120
Yes	2.9		22.9		0.04		2.4		0.3		3.1	
No	26.6		24.5		0.3		5.5		0.5		3.9	
Disease Triads												
ChoBpDiab		2.84, *p* = 0.093		0.04, *p* = 0.834		0.01, *p* = 0.933		22.67, *p* < 0.001		3.74, *p* = 0.054		1.08, *p* = 0.299
Yes	3.20		24.1		0.3		2.6		0.2		3.3	
No	26.3		24.3		0.3		5.5		0.5		3.9	
ChoBpOb		5.13, *p* = 0.024		13.43, *p* < 0.001		2.68, *p* = 0.102		14.78, *p* < 0.001		0.42, *p* = 0.518		0.02, *p* = 0.884
Yes	4.9		27.2		0.1		3.6		0.4		3.8	
No	24.6		23.8		0.3		5.4		0.5		3.8	
ChoDiabOb		0.37, *p* = 0.544		0.28, *p* = 0.596		2.82, p = 0.094		18.48, *p* < 0.001		1.30, *p* = 0.255		0.40, *p* = 0.528
Yes	2.0		25.0		0.0		2.2		0.2		3.5	
No	27.5		24.3		0.3		5.4		0.5		3.8	
BpDiabOb		4.34, *p* = 0.038		0.48, *p* = 0.491		3.89, *p* = 0.050		18.99, *p* < 0.001		2.28, *p* = 0.132		1.04, *p* = 0.309
Yes	2.3		23.5		0.1		2.5		0.2		3.3	
No	27.2		24.4		0.3		5.4		0.5		3.9	

Note: All percentages are weighted proportions

Disease Dyads: ChoBp = Hypercholesterolemia and Hypertension; ChoDiab = Hypercholesterolemia and Diabetes; ChoOb = Hypercholesterolemia and Obesity; BpDiab = Hypertension and Diabetes; BpOb = Hypertension and Obesity; DiabOb = Diabetes and Obesity

Disease Triads: ChoBpDiab = Hypercholesterolemia and Hypertension and Diabetes; ChoBpOb = Hypercholesterolemia and Hypertension and Obesity; BpDiabOb = Hypertension and Diabetes and Obesity; ChoDiabOb = Hypercholesterolemia and Diabetes and Obesity

### Sample characteristics

As described in Table 1, the sample was predominately White (75.6%), with the Southern region of the U.S. represented approximately twice the proportion of each of the other regions. Approximately 55.1% were female, 52.7% were married, and 73.2% were 50 years of age or older. About 76% had a high school or less education, and about one-quarter had graduated from college. Hypertension was most prevalent (73.2%), followed by hypercholesterolemia (61.1%), obesity (36.4%), and diabetes (22.6%). Approximately 37.2% of the participants had just one of the four chronic conditions, while 62.4% had more than one chronic disease.

### Bivariate and multivariate analysis of CAM use

#### Bivariate analysis

**Fig. 1:**
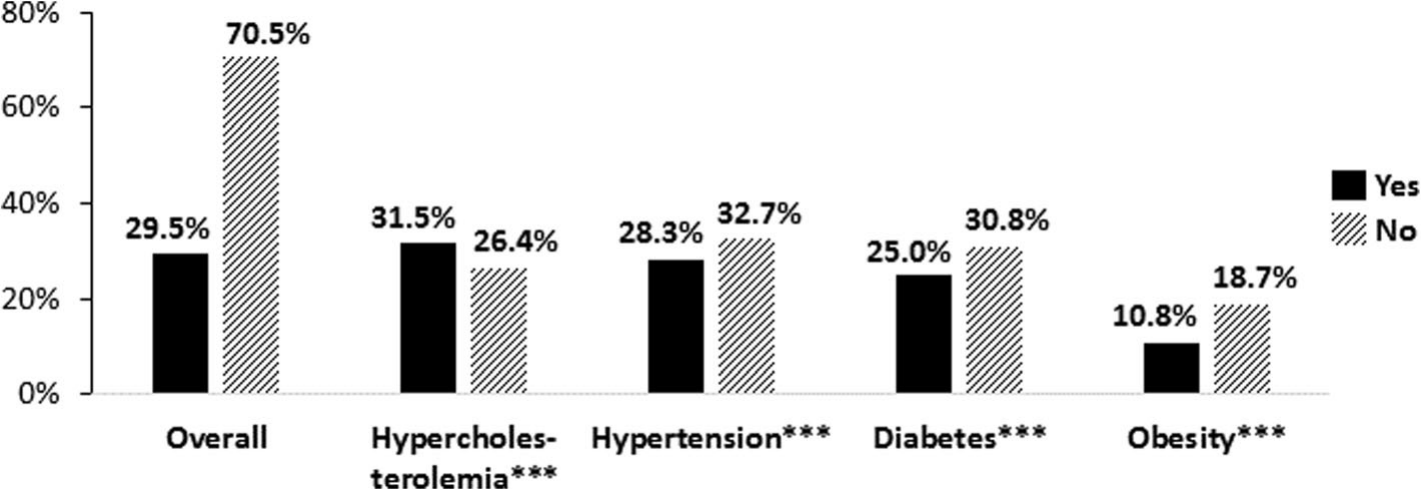
Overall and Individual Disease-Specific Rates of CAM Use. Overall: [Yes = Used CAM of any kind regardless of disease condition; No = Did not use CAM]; For individual disease conditions: [Yes = Used CAM and had the disease; No = Used CAM but did not have the disease]; Significance levels: *p < 0.05, **p < 0.01, ***p < 0.001

As noted in Fig. 1, overall 29.5% of the sample reported use of CAM of any kind. However, an examination of CAM use by disease status (see Table 2 and Fig. 1) showed lower proportions of participants with hypertension (28.3%), diabetes (25.0%), or obesity (10.8%) used CAM compared to those without these conditions (*p < 0.001*). Participants with hypercholesterolemia (31.5%) showed higher proportions of CAM use compared to those without this condition (*p < 0.001*). All the covariates except for sex were statistically associated with CAM use *(p < 0.05*). Additional results from the multivariate logistic regression analysis are reported in Table 2 with aORs controlling for potential confounding effects of the covariates in the model (e.g., education, sex, race/ethnicity, insurance status, etc.).

#### Multivariate analysis: Participants with one chronic condition

After controlling for confounding effects for those with one chronic condition, females were 25% more likely to use CAM than males (*p < 0.001*). Black/African Americans and the Non-Hispanic Other group were 54% and 39% less likely to report CAM use compared to Whites (*p < 0.001*), respectively. Compared to individuals with less than a high school education, high school and college graduates were 80% and 2.12 times more likely to report CAM use, respectively (*p < 0.001*). Participants with incomes in the range $35,000 to $49,999 or $50,000 to $74,999 were each 21% more likely to report CAM use compared to those with incomes less than $20,000 per year (*p < 0.05*). Finally, there was a 21% increased likelihood of using CAM for those participants residing in the Midwest or West, respectively, compared to Northeast residents (*p < 0.05*). For those with one chronic condition, there was no significant relationship in the adjusted odds of CAM use by age, marital status, having health insurance, or having a regular source of care.

#### Multivariate analysis: Participants with two or more chronic conditions

After adjusting for potential confounders for those with two or more chronic conditions, age, marital status, and having a regular source of care had no statistically significant effect on CAM use. Compared to Non-Hispanic Whites, Black/African Americans were 48% less likely to use CAM (*p < 0.001*), while Non-Hispanic Others were 25% less likely to report CAM use (*p < 0.05*). Those with health insurance and two or more conditions were 16% less likely to report CAM use compared to those without health insurance and two or more conditions (*p < 0.05*). Females were 29% more likely to report CAM use compared to males (*p < 0.001*).

Residents of the Midwest were 64% more likely to report CAM use (*p < 0.001*), while those from the West were 61% more likely to use CAM (*p < 0.001*), compared to those living in the Northeast. Compared to individuals with less than a high school education, high school and college graduates were 53% and 78% significantly more likely to report CAM use, respectively (*p < 0.001*). Finally, compared to participants making less than $20,000, participants with income ranges of $35,000 to $49,999 and $50,000 to $74,999 were 30% (*p < 0.001*) and 25% (*p < 0.01*) more likely to use CAM.

#### Multivariate analysis: CAM use by disease dyads and triads

**Fig. 2:**
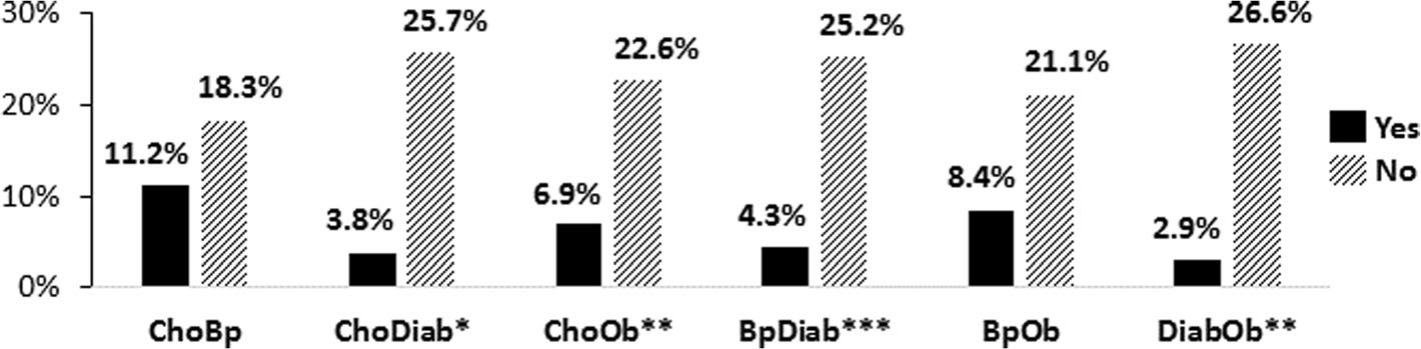
CAM Use by Disease Dyads. [Yes = Used CAM and had the disease dyad; No = Used CAM but did not have the disease dyad]; Disease Dyads: ChoBp = Hypercholesterolemia and Hypertension; ChoDiab = Hypercholesterolemia and Diabetes; ChoOb = Hypercholesterolemia and Obesity; BpDiab = Hypertension and Diabetes; BpOb = Hypertension and Obesity; DiabOb = Diabetes and Obesity; Significance levels: *p < 0.05, **p < 0.01, ***p < 0.001

Table 3 summarizes the proportions of CAM use by disease dyads and disease triads with respect to overall utilization and the specific CAM domains as operationally defined in this study. Diabetes co-occurring with hypercholesterolemia, hypertension, or obesity was significant for overall CAM use with rates ranging from 2.9 to 4.3% (*p < 0.05*). Those without these comorbidities using CAM had rates of 25.2% to 26.6%. Similarly, having both hypercholesterolemia and obesity yielded a significantly lower rate (6.9%) of CAM use compared to 22.6% among those using CAM who did not have this disease combination (*p < 0.01*). However, two disease dyads were not significant for CAM use among those with and without the disease combinations (i.e., hypertension and hypercholesterolemia; and hypertension and obesity). However, disease concordance with some of the highest proportions of CAM use (11.2% and 8.4%, respectively) was observed among persons within each of these groups, as noted in Fig. 2.

**Fig. 3:**
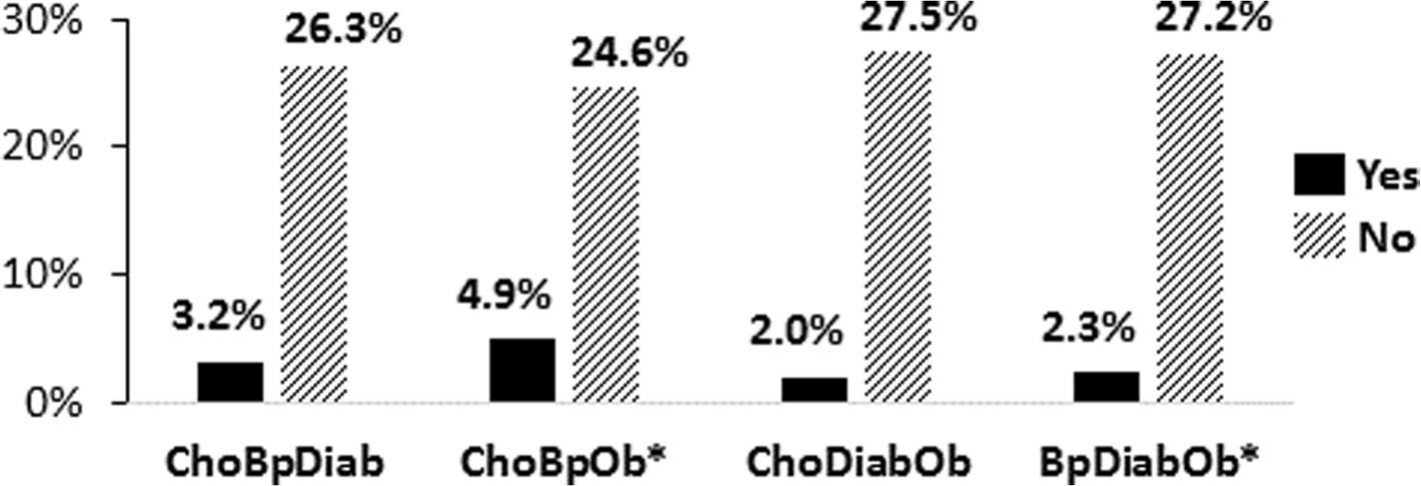
CAM Use by Disease Triads. [Yes = Used CAM and had the disease triad; No = Used CAM but did not have the disease triad]; Disease Triads: ChoBpDiab = Hypercholesterolemia and Hypertension and Diabetes; ChoBpOb = Hypercholesterolemia and Hypertension and Obesity; ChoDiabOb = Hypercholesterolemia and Diabetes and Obesity; BpDiabOb = Hypertension and Diabetes and Obesity; Significance levels: *p < 0.05, **p < 0.01, ***p < 0.001

Across the disease triads, as noted in Table 3, proportions of CAM use ranged from 2.0 to 4.9% for those with these comorbidities. However, as reflected in Fig. 3, with respect to disease triads, only two of the four triads (hypertension and obesity co-occurring with either hypercholesterolemia or diabetes) were significant (*p < 0.05*). Nearly 5% of persons with hypertension, obesity, and hypercholesterolemia reported CAM use compared to 24.6% who used CAM but did not have this disease combination (*p < 0.05*). Similarly, having hypertension, obesity, and diabetes was also significant for CAM use (2.3%) compared to 27.2% who did not have these conditions but used CAM (*p < 0.05*).

After adjusting for potential confounders for those with two or more chronic conditions, for the six dyad combinations (see Table 4), significant results for the adjusted odds ratios were only found for obese diabetic participants, who were 30% less likely to use CAM compared to those without these two chronic conditions (aOR = 0.70; 95% CI:0.55–0.88; *p < 0.01*). For the four disease triad combinations, significantly higher adjusted odds were only found for those participants with a concurrent diagnosis of hypercholesterolemia, diabetes, and obesity, who were 41% more likely to use CAM compared to those without these three chronic conditions (aOR = 1.41; 95% CI:1.02–1.94; *p < 0.05*). Additionally, those with hypercholesterolemia, hypertension, and obesity were 22% more likely to use CAM compared to those without these three comorbidities (aOR = 1.22; 95% CI:1.09–1.36; *p < 0.01*).

**Table 4:** Odds of CAM use among adults by chronic disease dyads and triads

Disease Combinations	Adjusted Odds of CAM Use	
	aOR [95% CI]	p-value
Disease Dyads		
ChoBp		
No (ref)	1.04[0.95–1.14]	0.351
Yes	1.00	–
ChoDiab		
No (ref)	0.92[0.75–1.11]	0.368
Yes	1.00	–
ChoOb		
No (ref)	1.08[0.96–1.21]	0.210
Yes	1.00	–
BpDiab		
No (ref)	1.06[0.91–1.24]	0.451
Yes	1.00	–
BpOb		
No (ref)	1.05[0.96–1.15]	0.309
Yes	1.00	–
DiabOb		
No (ref)	0.70[0.55–0.88]	0.003
Yes	1.00	–
Disease Triads		
ChoBpDiab		
No (ref)	1.00	–
Yes	1.11[0.94–1.32]	0.210
ChoBpOb		
No (ref)	1.00	–
Yes	1.22[1.09–1.36]	0.001
ChoDiabOb		
No (ref)	1.00	–
Yes	1.41[1.02–1.94]	0.037
BpDiabOb		
No (ref)	1.00	–
Yes	0.83[0.67–1.03]	0.083

Disease Dyads: ChoBp = Hypercholesterolemia and Hypertension; ChoDiab = Hypercholesterolemia and Diabetes; ChoOb = Hypercholesterolemia and Obesity; BpDiab = Hypertension and Diabetes; BpOb = Hypertension and Obesity; DiabOb = Diabetes and Obesity

Disease Triads: ChoBpDiab = Hypercholesterolemia and Hypertension and Diabetes; ChoBpOb = Hypercholesterolemia and Hypertension and Obesity; BpDiabOb = Hypertension and Diabetes and Obesity; ChoDiabOb = Hypercholesterolemia and Diabetes and Obesity

#### Multivariate analysis: CAM domain use by disease status

Across all four individual chronic conditions, manipulative methods had the highest rates of use (22–26%, *p < 0.05*). Alternative medical systems were also used by those with hypertension, hypercholesterolemia, or diabetes (3–4%, *p < 0.01*). For those with hypertension, diabetes, or obesity, approximately 3% to 5% used mind-body interventions (*p < 0.001*). Use of energy therapies (0.3%, *p < 0.001*) was only significant for those with hypertension, while use of biological treatments was not significant for any individual chronic condition. In general, the evidence suggests that there is greater use of manipulative methods, alternative medical systems, and mind-body interventions among participants with these individual chronic conditions. In addition, participants with hypertension utilized the greatest variety of treatments with significant usage across four of the five CAM domains, followed by participants with diabetes significantly utilizing treatments within three of the five CAM domains. Alternatively, participants with hypercholesterolemia or obesity significantly used CAM treatments within just two of the five CAM domains.

As noted in Table 3, there appears to be higher use of mind-body interventions and manipulative methods among participants with two comorbidities. Whereas mind-body interventions are consistently and statistically associated with all six disease dyads with utilization ranging from 2 to 4% (*p < 0.001*), manipulative methods were significant for three dyads with the highest proportions overall (23–27%, *p < 0.05*). The use of energy therapies was also significant for three of the disease dyads, but with lower proportions (0.2–0.3%, *p < 0.05*). The use of alternative medical systems was significant for just one disease dyad (3%, *p < 0.05*), as was the use of biological treatments (0.04%, *p < 0.05*). In the dyad participant populations, those with hypertension and diabetes utilized the greatest variety of treatments with significant usage across four of the five CAM domains, followed by participants with hypertension and hypercholesterolemia significantly utilizing treatments within three of the five CAM domains. Alternatively, participants with hypercholesterolemia and either diabetes or obesity, as well as obese diabetic participants significantly used treatments within two of the five CAM domains. Finally, obese hypertensive participants significantly used treatments within just one CAM domain.

Similar to the findings in the disease dyads, the use of mind-body interventions was significant (*p < 0.001*) across all disease triads with proportions ranging from 2 to 4%. However, the use of manipulative methods was only significant for persons with hypercholesterolemia, hypertension, and obesity (*p < 0.001*), with a large proportion of use (27%) for this group. The use of biological treatments, energy therapies, and alternative medical systems were not significant for any of the disease triads, with use trends all under 4%. In the triad participant populations, those with hypertension, hypercholesterolemia, and obesity utilized the greatest variety of treatments with significant usage across two of the five CAM domains, whereas all other triad participants significantly utilized treatments within just one of the five CAM domains.

## Discussion

An alternate study based on 2012 NHIS adult respondents with and without mental illness and two or more chronic physical conditions (including diabetes, hyperlipidemia, hypertension, and others) [17], similarly found higher patterns of CAM use across manipulative methods (15%) compared to mind-body interventions (6%) or alternative medical systems (4%) for those with physical comorbidities only. Manipulative methods, in particular, may appeal to users that prefer collaborative decision making with a supportive CAM practitioner [18]. Other researchers have documented that such therapeutic relationships can occur irrespective of the CAM treatment efficacy [5].

The observed diminished effect of disease triads on overall use of CAM as well as less use of specific modalities may reflect the fact that when individuals have more than two comorbidities, they are likely to be under strict care and management by a conventional health care provider. As such, these patients are likely to be on pharmaceutical agents or intentionally minimizing use of CAM products. With respect to manipulative methods, it is possible that patients with three chronic conditions are in such a debilitated state that engagement with these methods may not be possible.

### Limitations

Before discussing further implications of this study, several limitations must be noted. First, self-reported NHIS data are based on a sample of the population, and thus this study may be affected by sampling error and missing data. Second, CAM use trends may have changed since the publication of NHIS 2012 data set. In addition, the self-reported nature of the survey may have resulted in under-reporting of the various chronic diseases and CAM use. Nonetheless, the cross-sectional nature of the study allowed us to examine associations among sociodemographic factors, disease conditions, and use of a myriad of CAM therapies.

### Implications for family practitioners

This study has implications for the management of patients with chronic conditions, especially when these ailments co-exist. In this study, as summarized in Table 5, two CAM modalities (i.e., mind-body interventions and manipulative methods) dominated use patterns of participants with individual chronic conditions, as well as with disease dyads and triads composed of hypercholesterolemia, hypertension, diabetes, and obesity. These modalities have little interference and minimal to no side effects with conventional medicines that may be prescribed for these conditions. Alternatively, for some chronic diseases, alternative medical systems (3 individual disease conditions and 1 dyad), energy therapies (1 individual disease condition and 3 dyads), and biological treatments (1 dyad) were also significant choices for some participants.

**Table 5:** Statistically Significant CAM Modality Use by Disease Status

MIND-BODY INTERVENTIONS			MANIPULATIVE METHODS		
Hypertension*** Diabetes*** Obesity***	ChoBp*** ChoDiab*** ChoOb*** BpDiab*** BpOb*** DiabOb***	ChoBpDiab*** ChoBpOb*** ChoDiabOb*** BpDiabOb***	Hypercholesterolemia*** Hypertension** Diabetes*** Obesity*	ChoBp* ChoOb*** BpDiab*	ChoBpOb***
ALTERNATIVE SYSTEMS			ENERGY THERAPIES		
Hypercholesterolemia** Hypertension*** Diabetes**	BpDiab*		Hypertension***	ChoBp* ChoDiab* BpDiab*	
BIOLOGICAL TREATMENTS					
	DiabOb*				

Disease Dyads: ChoBp = Hypercholesterolemia and Hypertension; ChoDiab = Hypercholesterolemia and Diabetes; ChoOb = Hypercholesterolemia and Obesity; BpDiab = Hypertension and Diabetes; BpOb = Hypertension and Obesity; DiabOb = Diabetes and Obesity

Disease Triads: ChoBpDiab = Hypercholesterolemia and Hypertension and Diabetes; ChoBpOb = Hypercholesterolemia and Hypertension and Obesity; BpDiabOb = Hypertension and Diabetes and Obesity; ChoDiabOb = Hypercholesterolemia and Diabetes and Obesity

Significance levels: *p < 0.05, **p < 0.01, ***p < 0.001

## Conclusions

As integrative medicine becomes commonplace, family practitioners will play a pivotal role in educating patients on the benefits and potential harm of CAM products [19, 20]. This is especially true as more patients require maintenance medications that may increase the risk for multi-drug or herb-drug interactions or nullify the sometimes narrowly windowed therapeutic effects of the pharmaceutical agents indicated for these conditions [7, 21]. Our study provides prevalence data concerning the use of CAM modalities among persons with a variety of chronic condition dyads and triads, and these disease combinations have gone largely understudied in the scientific literature with respect to specific CAM domain usage rates. Patients with multiple comorbidities use various non-conventional approaches, and as such, it is important for health care providers at every level to proactively probe patients on the use of CAM products and or services and to offer personalized information about the possible risks, benefits, and potential implications of using CAM. Indeed, research has consistently shown patients do not always voluntarily divulge information on CAM use to providers [5, 22, 23]. This study focused on four chronic conditions due to their prevalence in the general population. As clinical practice guidelines and life-style recommendations for multimorbid patients continue to be developed to include CAM, future research on these and other comorbidities may contribute to improved health care utilization and patient outcomes.

## Abbreviations

aOR: Adjusted odds ratio
CAM: Complementary and alternative medicine
CI: Confidence interval
NCHS: National Center for Health Statistics
NHIS: National Health Interview Survey
OR: Odds ratio
